# Potential Cellular Functions of Epstein-Barr Nuclear Antigen 1 (EBNA1) of Epstein-Barr Virus

**DOI:** 10.3390/v5010226

**Published:** 2013-01-16

**Authors:** Danielle Westhoff Smith, Bill Sugden

**Affiliations:** Department of Oncology, McArdle Laboratory for Cancer Research, Madison, WI 53706, USA; E-Mail: dwesthoff@wisc.edu

**Keywords:** Epstein Barr Virus, Epstein Barr Virus nuclear antigen one, human tumor virus, cancer

## Abstract

Epstein-Barr Nuclear Antigen 1 (EBNA1) is a multifunctional protein encoded by EBV. EBNA1’s role in maintaining EBV in latently proliferating cells, by mediating EBV genome synthesis and nonrandom partitioning to daughter cells, as well as regulating viral gene transcription, is well characterized. Less understood are the roles of EBNA1 in affecting the host cell to provide selective advantages to those cells that harbor EBV. In this review we will focus on the interactions between EBNA1 and the host cell that may provide EBV-infected cells selective advantages beyond the maintenance of EBV.

## 1. Introduction

Epstein-Barr Virus (EBV) is causally associated with a variety of B-cell and epithelial cell malignancies including Burkitt’s and Hodgkin’s lymphomas, post-transplant lymphoproliferative disease, and gastric and nasopharyngeal carcinomas [1]. In its latently proliferating state, EBV expresses variable subsets of viral genes. The most restricted subset of viral genes expressed during latency consists of one set of viral miRNAs (BARTs), the EBERs (two Epstein-Barr small noncoding RNAs), and the only latent viral protein known to be consistently expressed across all EBV-associated malignancies, Epstein-Barr Nuclear Antigen 1 (EBNA1). EBV depends on EBNA1 for the critical processes it mediates in the viral life cycle.

EBNA1 is a homo-dimeric, DNA-binding protein that binds site-specifically to a DNA sequence 16 bp in length [2,3] via its DNA-binding and dimerization domain located at the C-terminal end. By binding to clusters of EBNA1-binding sites within the EBV latent origin of replication (*OriP*), EBNA1 mediates EBV’s genome synthesis [4] and the nonrandom partitioning of newly synthesized viral plasmids to daughter cells during mitosis [5,6].

EBNA1 also functions as a viral transcriptional transactivator. When bound to the family of repeats (FR), EBNA1 enhances transcription from Cp, a downstream promoter active during the latent phase of the viral life-cycle [7,8]. Similarly, EBNA1 bound to FR enhances transcription of the viral gene, Latent Membrane Protein 1 (LMP1), from a distance of 10 kbp across the circularized ends of the viral genome [9]. Additionally, binding of FR by EBNA1 enhances transcription from heterologous promoters [10]. Through its transcriptional transactivation function, EBNA1 enhances transcription of the latent genes required for the induction and maintenance of proliferation in EBV infected cells. However, EBNA1 may also negatively regulate transcription. For example, EBNA1 has been shown to bind site-specifically to two sites at position +10 of the viral promoter Qp and inhibit its own expression [11,12]. The mechanism of this inhibition remains enigmatic, though.

**Figure 1:**
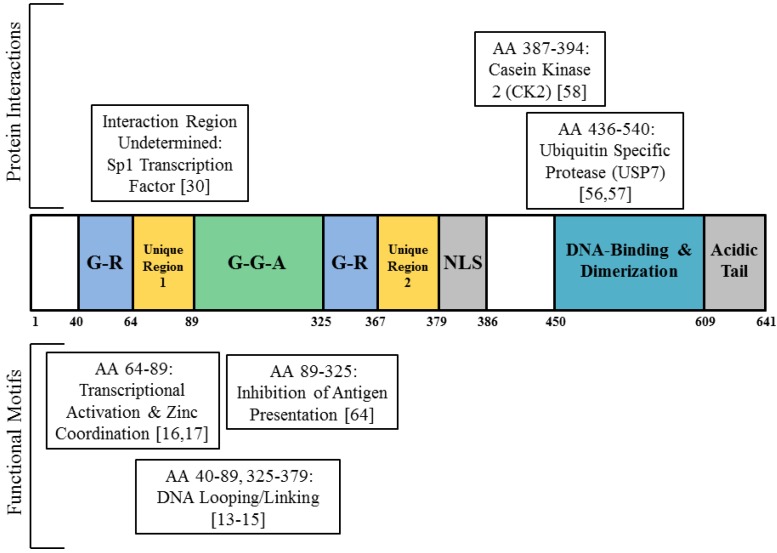
A Structural Representation of Epstein-Barr Nuclear Antigen 1 (EBNA1) with the Protein Interacting Domains and Functional Motifs discussed in this review. The Brackets give citations identifying these domains and motifs. The Amino Acid (AA) positions of each domain are noted. G-R notes the glycine-arginine rich regions within the DNA Looping/Linking regions. G-G-A notes the glycine-alanine repeat region. NLS notes the Nuclear Localization Signal.

The mechanism by which EBNA1 activates transcription is not well defined. It is known that EBNA1 can link regions of DNA to which it binds, and in particular can form a loop between FR and the Dyad of Symmetry (DS) [13,14]. The regions necessary for this activity are amino acids 40–89 and 329–378 (Figure 1) [15]. The ability of EBNA1 to link DNA correlates with its support of replication and transcription [16]. Also, the Unique Region 1 (UR1) region of EBNA1, amino acids 64–89, contains two conserved cysteine residues that coordinate zinc (Figure 1). Chelation of cellular zinc or alanine substitutions of these conserved cysteine residues reduces the ability of EBNA1 to activate transcription [17].

While it is clear that EBNA1 serves necessary roles for the maintenance of the EBV genome and for the expression of other viral proteins in latently infected cells, EBNA1 may also interact with the host cell to provide selective advantages to those cells that harbor EBV. In this review we will focus on the ways in which EBNA1 may provide EBV-infected cells selective advantages beyond ensuring the maintenance of EBV, and propose additional experiments needed to identify the functional interactions between EBNA1 and the host cell.

## 2. EBNA1 Binds the Cellular Genome

EBNA1’s ability to regulate viral gene expression and mediate the replication and partitioning of the viral genome depend upon its ability to bind site-specifically to the viral genome through its C-terminal DNA-binding and dimerization domain. Multiple studies have shown EBNA1 also binds site-specifically to the cellular genome, raising the possibility that EBNA1 does so to benefit EBV. The first study to assess this binding found low-affinity binding sites, but concluded that because of their affinity, EBNA1 was unlikely to bind these sites functionally [18]. However, subsequent work has identified phenotypes of EBNA1 in the absence of other viral genes that could result from EBNA1’s binding cellular DNA. For instance, over-expression of EBNA1 has been found to be cytotoxic in both human epithelial cells [19] and B-lymphoid cells [20] in the absence of EBV. Likewise, DNA-binding derivatives of EBNA1 when over-expressed in primary effusion lymphoma cells (PELs) were found to inhibit their colony formation independently of EBV. These lymphomas do carry a related Herpesvirus, Kaposi’s Sarcoma-Associated Herpesvirus (KSHV). The colonies that did form contained mutations in the EBNA1 derivatives blocking their DNA-binding activity, providing evidence that the observed phenotype resulted from these EBNA1 derivatives binding either KSHV DNA or cellular DNA [21].

EBNA1 chromatin immunoprecipitation (ChIP) and sequence analysis with either human promoter arrays or deep sequencing have now identified and defined cellular sites to which EBNA1 binds; some being bound with high affinities [22,23,24]. In these studies, varying methodologies were used to define EBNA1 DNA-binding consensus sequences. For instance, a Position Weighted Matrix (PWM) was developed using a subset of these cellular sites which had been confirmed by Electro Mobility Shift Assays (EMSA) to be bound by EBNA1, as well as non-redundant EBNA1-binding sites within the EBV genome. This PWM predicted binding sites in a virus related to EBV, lymphocrytovirus ceHV-15, which were shown to bind EBNA1 and support synthesis of plasmids containing those sites [22], proving the ability of this derived PWM to identify DNA sequences functionally bound by EBNA1. Additional studies derived multiple consensus sites using solely those EBNA1-associated cellular DNA sequences they identified [23,24]. Though each study derived their EBNA1 consensus motifs using separate sets of input sequences, the consensus motifs generated by these EBNA1 ChIP experiments all differ from the consensus sequence for EBNA1’s binding sites within the EBV genome. The diversity of these consensus sequences reflects the degeneracy of sequences that can be bound by EBNA1 and the range of affinities of EBNA1 for binding them. That EBNA1 has evolved to bind such a diverse set of sequences in addition to the sequences within the EBV genome supports the hypothesis that by binding to cellular sites, EBNA1 performs some function(s) beneficial to EBV’s life cycle.

However, identifying any function of EBNA1 mediated by its being bound to these cellular sites has proved difficult. For example, clusters of high affinity EBNA1-binding sites, resembling FR, have been identified in the human genome ([24,25], and our own unpublished observations). Neither ORC2 or MCM proteins were found to bind at these clusters [24], indicating these sites do not function as origins of replication. This finding may not be surprising, though; EBNA1 can only recruit cellular replication proteins to pairs of binding-sites separated exactly by 21 base pairs [26,27].

That EBNA1 binds to sites within the human genome makes it likely that by binding to cellular sites, EBNA1 regulates cellular gene transcription. For instance, EBNA1 is able dose-dependently to activate transcription from an integrated copy of FR [28]. No evidence has been found for EBNA1 activating transcription when bound to nearby FR-like clustered sites such as Fam55 B and D, though [24]. Additional studies have tested whether EBNA1 affects the expression of other cellular genes near to DNA sites where EBNA1 is bound, and have produced mixed results. A study in which the cellular promoters bound by EBNA1 were cloned upstream of the luciferase gene failed to show enhanced transcription in the presence of EBNA1 [22]. On the other hand treating EBV-positive, Raji cells with siRNA to EBNA1 reduced mRNA levels of a subset of cellular genes with EBNA1-bound promoters. Within this subset, the expression of four cellular genes was increased by exogenous expression of EBNA1 in the EBV-negative Burkitt’s Lymphoma cell line DG75 [24]. Another study found an overlap with a set of genes with promoters associated with EBNA1 that were also differentially regulated in EBNA1-positive BJAB cells [23]. In addition, d’Herouel *et al.* predicted EBNA1-binding sites within the ATF2 and c-Jun promoters [25], while a separate study found EBNA1 to be associated with the promoters of ATF2 and c-Jun by ChIP. This association correlated with a two-fold and four-fold increase in mRNA levels of ATF2 and c-Jun, respectively [29], though it has not been shown that EBNA1 binds directly to these promoters. Nor is EBNA1 predicted to bind near the promoters using the PWM developed by Dresang *et al.* (Table 1). EBNA1 does increase the expression of Survivin mRNA in Burkitt’s lymphoma cells, and is associated with the Survivin promoter. However, this interaction is mediated through the transcription factor Sp1 (Figure 1) [30].

**Table 1:** **Genes whose transcription is reported to be regulated by EBNA1.** The Position Weighted Matrix (PWM) developed by Dresang *et al.* [22] predicts EBNA1-binding sites associated with only a subset of genes with increased mRNA levels in the presence of EBNA1. The PWM was used to predict EBNA1-binding sites within 20 kilobase pairs (kbp), both 5’ and 3’, of the transcriptional start site of the listed gene. The position P-value is a measure of the probability that a random sequence would match the consensus site as well as the sequence of interest [22,31]. The position P-value for the lowest affinity EBNA1-binding site within the EBV genome to which EBNA1 is functionally bound (Rep* Site 2) is 7.7E-05. *MYO1C has three alternative transcription start sites.

Gene	Binding sitePosition P-value	Distance fromTranscription Start	Reference
Nox 2	4.79E-05	16 kbp 5'	[32]
HDAC3	-	None within 20 kbp	
MAP3K1	4.13E-05	13.5 kbp 5'	
PBX2	-	None within 20 kbp	[24]
MYO1C-1*	2.93E-06	6.9 kbp 3'	
MYO1C-2*	-	4.9 kbp 5'	
MYO1C-3*	-	5.9 kbp 3'	
c-Jun	-	None within 20 kbp	[25,29]
ATF2	-	None within 20 kbp	
STAT 1	9.37E-06	11 kbp 3'	[33]
CCL18	-	None within 20 kbp	
CCL3	-	None within 20 kbp	[23]
CCL4	-	None within 20 kbp	
TIAL1	9.10E-06	None within 20 kbp	

Together these studies are consistent with the hypothesis that EBNA1 can regulate the transcription of some genes. The presence of an EBNA1-binding site is not sufficient to predict EBNA1’s regulation of nearby gene expression, though. Neither can the PWM developed from one study predict EBNA1-bound sites identified by other studies (Table 1). Furthermore, among those genes that have been reported to be regulated by EBNA1 via EBNA1’s increasing the levels of their mRNAs, the position of the EBNA1-binding site gives little information on how EBNA1 might affect gene expression (Table 1). The position of EBNA1-binding sites relative to the transcriptional start sites of these genes is not consistent. Therefore, it would be useful to derive a new EBNA1 PWM from functionally bound sites as more EBNA1-binding sites from which EBNA1 regulates cellular gene expression are experimentally confirmed. This consensus sequence would prove a useful tool in directing the search to identify additional cellular sites functionally bound by EBNA1. It has been speculated that EBNA1 acts as a “pioneer transcription factor”, that DNA looping induced by EBNA1 re-organizes cellular chromatin structure to allow progressive binding of cellular transcription factors [34]. EBNA1 on binding the sites with in FR, does increase the affinity of EBNA1 for sites in the nearby DS element to which it loops *in vitro* [13]. In addition to DNA looping induced by EBNA1 homotypic interactions, it is possible that DNA looping may be induced by an interaction between EBNA1 and other chromatin and nucleosome-binding proteins such as Brd4, PRMT5, TAF-1β, NAP-1, or Nucleophosmin with which EBNA1 has been reported to interact [35,36,37,38]. Such long-range interactions between EBNA1 and cellular chromatin-binding proteins may affect the transcription of cellular genes, possibly explaining those instances where an EBNA1-binding site is not predicted near the transcriptional start site of genes that are regulated transcriptionally in EBNA1’s presence (Table 1), or contribute to EBNA1’s repression of the viral Qp promoter. However, it is yet to be demonstrated that EBNA1 binds and re-organizes condensed chromatin to allow further binding of transcription factors and transcriptional regulation, nor that EBNA1 induces long-range looping of cellular DNA *in vivo*. Nevertheless, gaining an understanding of functionally bound sites and the genes EBNA1 regulates when bound to them will be necessary to understand any selective advantages EBNA1 provides host cells through its presumptive regulation of cellular genes.

## 3. EBNA1 as a Potential Regulator of Signaling Pathways

EBV must contribute one or more selective advantages to proliferating cells to be maintained in them as a plasmid [6]. More narrowly, the properties of some EBV-caused tumors are consistent with EBNA1 contributing significantly to maintaining those tumors. For example, some Burkitt’s Lymphomas express EBNA1 as their only EBV-encoded protein and EBV has been shown explicitly to provide these tumors selective advantages [39,40] One certain advantage EBNA1 provides these cells is its maintenance of the viral genome. A second potential role for EBNA1 in affecting its host cell would be for it to regulate signaling pathways to afford its host one or more selective advantages. This possibility has been explored by establishing a derivative of an epithelial cell line, Ad/Ah, that expresses EBNA1 and searching for transcriptional changes in the EBNA1-positive derivative relative to the parental cells carrying an empty vector [33]. This approach has led to three experimental questions: 1. Does any regulation by EBNA1 depend on the type of its host cell? 2. Do the levels of expression of EBNA1 in a particular cell affect EBNA1’s capacity to regulate signaling pathways? 3. Do additional EBV genes affect EBNA1’s capacity to regulate signaling pathways? We shall review the observations, their interpretations, and then note additional experiments needed to determine if EBNA1’s possible regulation of signaling pathways contributes to its affecting tumors caused by EBV.

Microarray analysis of Ad/Ah cells identified 162 genes whose mRNA levels differed when these cells expressed EBNA1 compared to the cells carrying an empty vector control [33]. Among these transcriptional changes, STAT1 was increased in EBNA1-positive cells, which correlated with an increase in their expression of MHC Class I and MHC Class II molecules. Though STAT1 has a predicted EBNA1-binding site near it, the site is 11 kpb 3’ to STAT1’s transcriptional start site (Table 1). Whether EBNA1 directly activates STAT1 transcription is not yet known. This same microarray analysis also found that the mRNA level of a TGFβ1-regulated gene, βig-h3, was reduced in EBNA1’s presence, indicating EBNA1 might impair TGFβ1 signaling. Though EBNA1 had no effect on TGFβ1 mRNA or protein levels, the presence of EBNA1 was found to reduce the half-life of SMAD2, a mediator of TGFβ1 signaling, from 5 hours to 2.5 hours [33]. A study using Hodgkin’s Lymphoma cells likewise found a reduced half-life of SMAD2 and found also the level of the TGFβ1 responsive gene, Protein Tyrosine Phosphotase Receptor K (PTPRK), was reduced in EBNA1’s presence [41].

Two additional studies using an *in silico* approach have found that 15% and 20% of the promoters of cellular genes differentially regulated in the presence of EBNA1 in Ad/AH cells contained DNA-binding motifs for the transcription factors NF-κβ [42] and AP-1 [29], respectively. These studies find that EBNA1 enhances AP-1 activity by inducing the transcription of the AP-1 subunits c-Jun and ATF2 [29], but inhibits NF-κβ activity; possibly by decreasing the phosphorylation of the NF-κβ activating Ikkα/β kinase complex [42].

These findings have been interpreted to indicate that EBNA1 modulates a subset of the host cell’s signaling pathways, and thereby contributes to the survival and proliferation of EBV-infected cells. While this interpretation may prove correct, some of the findings are currently too tangential to support it. For example, though treating a Hodgkin Lymphoma cell line with siRNAs to PTPRK increases cell viability [41], the expression of EBNA1 in an EBV-positive Hodgkin Lymphoma cell seems unlikely to provide such a selective advantage because EBV fails to provide these cells sufficient selective advantages to be maintained in them as they proliferate in culture (ibid).EBV plasmids are generally lost from explanted Hodgkin’s Lymphomas. It is also clear that expression of additional EBV genes can affect the signaling pathways suggested to be regulated by EBNA1. For instance, the viral gene LMP1, via its CD40-like carboxy-terminal domain, activates the NF-κβ, STAT1, and AP1 signaling pathways to drive cellular proliferation and inhibit apoptosis [43]. Teasing out whether EBNA1 acts in opposition to, or coordinates with additional EBV latent genes, such as LMP1, will be necessary to understand EBNA1’s contribution to EBV’s providing cells a selective advantage via its regulation of signaling pathways.

The role of EBNA1 in EBV’s latent infection makes it particularly difficult to identify a possible role for EBNA1 in regulating cellular signaling pathways. Studies of EBNA1 in the absence of other viral genes miss their functional interactions while modulating levels of EBNA1 in latently infected cells affects the levels of other expressed viral genes. It is therefore important to use sequential analyses of EBNA1 alone and in the context of the viral genome in cells normally infected by EBV to elucidate any role EBNA1 may have in regulating cellular signaling pathways.

## 4. EBNA1 as an Oncogene

Because some Burkitt’s Lymphomas express EBNA1 as their only-EBV encoded protein, attempts have been made to determine if EBNA1 behaves as an oncoprotein. In particular two groups have arrived at opposite conclusions using transgenic mice to learn if EBNA1 can be tumorigenic in mice. B-cell directed expression of EBNA1 increased the prevalence of B cell lymphomas in two C57BL/6 mouse lines [44]. Pre-neoplastic lesions in the EBNA1 transgenic mice had increased expression of bcl-xL and the recombination activating genes (RAG) 1 and 2 [45], and co-expression of Myc could synergize with EBNA1 to shorten the time to tumor development [46]. Yet, two additional, seemingly parallel, studies using FVB and C57BL/6 mice did not find evidence for increases in B cell lymphomas in the EBNA1-transgenic mice [47,48]. The study by Wilson *et al*. generated 10 lines of EBNA1 transgenic C57BL/6 mice, one line giving rise to lymphomas in 100% of the mice by 12 months and another line with lymphomas occurring in only 29% of mice by 24 months [44]. However, no lymphomas were found among three lines of EBNA1 transgenic FVB mice by 26 months [47]. The frequency with which distinct transgenic lines do yield tumors (2 of 13) indicates that EBNA1 is not an oncogene as potent as c-Myc, for example. It is likely that other mutations, the site of transgene insertion, or that a narrow range of EBNA1 expression are required in addition to EBNA1 for the tumor development in transgenic mice. The mechanism by which EBNA1 does contribute to lymphomas in some transgenic lines has yet to be elucidated.

In cell culture EBV infection and constitutive or conditional expression of EBNA1 in Burkitt’s Lymphoma cell lines led to increased levels of Reactive Oxygen Species (ROS) and genomic instability [32,49]. These increased levels resulted from increased expression of the catalytic subunit of the NADPH oxidase, Nox2, in cells that express EBNA1 [32]. Similarly, the expression of EBNA1 in CNE2 cells increased expression of Nox2 and ROS [50]. It is unlikely that EBNA1 affects the transcription of Nox2 directly (Table 1). The increase in ROS associated with expression of EBNA1 paralleled an increase in telomere instability [51]. These results have led to the suggestion that EBNA1 acts as an oncoprotein by contributing to the genomic instability and oxidative stress observed in EBV-infected cells [52,53].

It is not now clear, however, that genomic instability other than Ig/c-Myc translocations mediated by A.I.D [54] is common in primary Burkitt’s Lymphoma. The Mitelman Database of Chromosomal Alterations in Cancer found that the karyotypes of Burkitt’s Lymphoma tumors have few to no rearrangements other than the Ig/cMyc translocations whether they are EBV-positive or not [55]. Furthermore, though EBV infection of B-cells induces a DNA damage response, this response is transient and is attenuated by the time EBV infected cells have undergone approximately seven population doublings [56]. Though this early DNA damage response may lead to non-clonal chromosomal alterations, normal, EBV-positive B-lymphoid cell lines (LCLs) maintain a stable karyotype [57] and have a low level of ongoing DNA damage response [56]. If EBNA1 does induce genomic instability by increasing ROS, this induction may be an effect of EBNA1 being the sole EBV protein expressed in Burkitt’s Lymphoma, and EBV’s other latent genes may inhibit this effect. If ongoing ROS production by EBNA1 is occurring in Burkitt’s Lymphoma, a study of primary Burkitt’s Lymphomas is needed to measure the prevalence of non-clonal chromosomal alterations in these tumors.

A defining characteristic of Burkitt’s Lymphomas is the constitutive activation of c-Myc via translocations that bring it under control of the *Ig* locus. Yet, though c-Myc expression induces cell growth and proliferation, making it a potent oncogene, c-Myc expression also triggers cellular senescence and apoptosis (reviewed in [58]). Thus, for Burkitt’s Lymphomas to continue to proliferate, the apoptotic pathway must be inhibited. EBV encodes multiple genes that inhibit apoptosis, including BHRF1 and BALF1 which are related to the anti-apoptoic protein BCL2 [59] and EBNA3A and 3C which can inhibit pro-apoptotic Bim [60,61,62]. EBNA1 may also inhibit apoptosis. By binding to USP7/HAUSP (Figure 1), a cellular ubiquitin-specific protease, EBNA1 is suspected to cause p53 destabilization in EBV-positive cells [35,63]. EBNA1 is also reported to interact with the protein kinase CK2 (Figure 1) and through this interaction disrupt PML nuclear bodies and degrade PML proteins [35,64,65]. By destabilizing both p53 and PML proteins, EBNA1 decreases sensitivity to DNA damage and inhibits apoptosis in cells that are treated with DNA damaging agents [63,64,65].

Yet, in contrast to these studies, in LCLs treated with DNA damaging agents, p53 is phosphorylated and accumulates, and the cells die by apoptosis [66,67], indicating that the p53 pathway is functional in LCLs. In contrast, p53 is mutated in approximately 30% of Burkitt’s Lymphoma biopsies ([68,69] and references therein). Frequently, those Burkitt’s Lymphoma that do carry wild-type p53 either overexpress MDM2, or have lost expression of p14ARF [69]. These findings indicate that during the evolution of Burkitt’s Lymphoma tumors, the p53 pathway is inhibited through genetic selection, rather than EBNA1’s direct repression of this pathway. Though EBNA1 may have some effect on p53 and PML proteins that contributes to the inhibition of apoptosis, given the frequency with which this pathway is mutated in Burkitt’s Lymphoma, and the known roles of other viral genes in disrupting apoptosis, EBNA1’s contribution to inhibiting apoptosis now seems likely to be limited.

## 5. EBNA1 Inhibits its Own Antigen Presentation

While examples of EBNA1’s regulating expression of cellular genes transcriptionally remain incomplete, EBNA1 does affect its own recognition as an antigen. The expression of EBNA1 in infected cells does not lead to those cells being recognized efficiently by cluster of differentiation 8 (CD8)-positive cytotoxic T-cells. A stretch of glycine-alanine repeats in this viral protein limits its presentation as an antigen on major histocompatibility complex (MHC) class I molecules (Figure 1) [70]. Instead, EBNA1 stimulates CD4-positive T-cells when presented on infected B-cells by MHC class II molecules [71,72,73]. Some of these epitopes are generated via autophagy, and EBNA1 can be found in double membrane vesicles, consistent with its localization to autophagosomes [74,75,76]. Suppression of autophagy with small interfering RNAs (siRNAs) that target mediators of autophagy, ATG12 or ATG7 (ATG refers to “autophagy related”) inhibits stimulation of CD4-positive T-cells after their exposure to EBV-positive cells [74,75]. Similarly, CD4-positive T-cells remain unstimulated when the acidification of autophagolysosomes in infected cells is inhibited [75]. Despite its immunogenicity, EBNA1 is poorly presented on MHC II molecules, perhaps because it spends little time in the cytoplasm [73,74,77,78]. How the presentation of EBNA1 to CD4-positive T-cells contributes to EBV’s life-cycle is unclear. However, its poor presentation by MHC class II molecules surely contributes to some EBV-positive tumors, such as canonical Burkitt’s lymphomas in which EBNA1 is the only viral protein expressed, escaping the hosts’ immune response.

## 6. Conclusions

EBNA1 contributes multiple functions that support the latent phase of EBV’s life cycle, including the synthesis and partitioning of the viral genome in proliferating cells and the regulation of expression of some viral genes. EBNA1 also binds sequence specifically to many sites in the human genome in infected cells so that it has the potential to regulate some cellular genes directly if it binds productively near them, or indirectly by means of genes it may directly regulate. Many candidates for being regulated directly or indirectly by EBNA1 have been suggested, but none has been unequivocally identified. Other kinds of phenotypes have been attributed to EBNA1, including oncogenicity in transgenic mice and stimulation of ROS in human cells. The generality of these phenotypes among EBV infected cells and mechanisms that possibly underlie them are not yet established. Even though it seems likely that EBNA1 will affect host cells through its binding specifically to sites in the human genome, and much work has been done to explore this likelihood, no compelling examples for such an effect have been fully characterized. If such a role for EBNA1 does exist, it will need to be documented both alone and in the intact viral genome within cells to uncover its significance for EBV’s life-cycle.
